# Impact of radiation dose reduction and iterative image reconstruction on CT-guided spine biopsies

**DOI:** 10.1038/s41598-023-32102-9

**Published:** 2023-03-28

**Authors:** Karolin J. Paprottka, Karina Kupfer, Vivian Schultz, Meinrad Beer, Claus Zimmer, Thomas Baum, Jan S. Kirschke, Nico Sollmann

**Affiliations:** 1 Department of Diagnostic and Interventional Neuroradiology, School of Medicine, Klinikum rechts der Isar, Technical University of Munich, Munich, Germany; 2 TUM-Neuroimaging Center, Klinikum rechts der Isar, Technical University of Munich, Munich, Germany; 3 Department of Diagnostic and Interventional Radiology, University Hospital Ulm, Ulm, Germany

**Keywords:** Medical imaging, Computed tomography

## Abstract

This study aimed to systematically evaluate the impact of dose reduction on image quality and confidence for intervention planning and guidance regarding computed tomography (CT)-based intervertebral disc and vertebral body biopsies. We retrospectively analyzed 96 patients who underwent multi-detector CT (MDCT) acquired for the purpose of biopsies, which were either derived from scanning with standard dose (SD) or low dose (LD; using tube current reduction). The SD cases were matched to LD cases considering sex, age, level of biopsy, presence of spinal instrumentation, and body diameter. All images for planning (reconstruction: “IMR1”) and periprocedural guidance (reconstruction: “iDose4”) were evaluated by two readers (R1 and R2) using Likert scales. Image noise was measured using attenuation values of paraspinal muscle tissue. The dose length product (DLP) was statistically significantly lower for LD scans regarding the planning scans (SD: 13.8 ± 8.2 mGy*cm, LD: 8.1 ± 4.4 mGy*cm, *p* < 0.01) and the interventional guidance scans (SD: 43.0 ± 48.8 mGy*cm, LD: 18.4 ± 7.3 mGy*cm, *p* < 0.01). Image quality, contrast, determination of the target structure, and confidence for planning or intervention guidance were rated good to perfect for SD and LD scans, showing no statistically significant differences between SD and LD scans (*p* > 0.05). Image noise was similar between SD and LD scans performed for planning of the interventional procedures (SD: 14.62 ± 2.83 HU vs. LD: 15.45 ± 3.22 HU, *p* = 0.24). Use of a LD protocol for MDCT-guided biopsies along the spine is a practical alternative, maintaining overall image quality and confidence. Increasing availability of model-based iterative reconstruction in clinical routine may facilitate further radiation dose reductions.

## Introduction

Advances in imaging technologies enable a better differentiation between benign and malignant bone lesions, detection of smaller bone lesions by radiologists, and a better diagnosis of different inflammatory processes^1,2^. However, the comparatively low specificity of imaging modalities for correct diagnosis still demands a histopathologic confirmation in indeterminate cases^3–5^. For obtaining a representative tissue sample, a biopsy via an open surgical access is still considered the reference standard^6^. However, this open surgical access also has disadvantages, such as high time expense, high cost, prolonged hospitalization, and risks of general anesthesia and major follow-up surgery (e.g., due to complications)^7^.

As an alternative, imaging-guided percutaneous biopsy is a well-described and successful technique for yielding histopathologic diagnoses^8,9^. Specifically, it can yield an accuracy of over 94% in determining benign, inflammatory, or malignant etiologies^10^. In this regard, the Infectious Diseases Society of America officially recommends computed tomography (CT)-guided biopsies via a percutaneous approach as a first attempt in many cases of suspected discitis.

Guidance using CT is often considered for precise localization of a lesion before and during biopsy^7,8,11,12^. It gives the interventionalist not only a detailed view of the anatomic circumstances for biopsy planning and execution, but also allows for confirmation of the correct needle placement within the area of concern during the procedure. At many institutions, CT guidance is the primary method of choice for biopsies of osseous lesions within the vertebrae or with regard to suspected infections along the spine^3,7,13^, owing to high availability, comparatively low cost, high spatial resolution, and relatively few procedure-limiting contraindications. Although CT guidance is frequently used for various procedures, there is concern over the amount of radiation exposure for the patient. Radiation dose reduction is commonly used in diagnostic scans especially for pediatric patients^14,15^. Additionally, it is also a relevant issue for patients who receive multiple scans due to acute disease follow-up exams, chronic diseases, and screening purposes^16–19^. Hence, there are many studies concerning the safety and efficacy of spine biopsies^5,7,20^. However, with the wider availability of modern model-based iterative reconstruction techniques, even further radiation dose reduction could become possible when combined with systematic lowering of the tube current. Specifically, no studies with a matched-pair design are available regarding dose reductions for planning and performing CT-guided biopsies at the spine with a focus on image quality and confidence of the interventionalist regarding the procedure.

The purpose of our retrospective study was to demonstrate that a low-dose (LD) protocol for CT-guided percutaneous biopsies of the vertebral bone or intervertebral discs is a practical alternative to standard-dose (SD) approaches, maintaining overall image quality and confidence for the interventionalist for planning and performing a safe procedure at reduced ionizing radiation doses.

## Material and methods

### Study cohort

We retrospectively reviewed CT-guided intervertebral disc or vertebral bone biopsies, which were performed with two different dose levels: SD and LD scans for planning and procedure performance. A general adjustment of our institutional CT protocols took place in October 2020, hence all LD scans that were included in our study were acquired between November 2020 and June 2021, while all SD scans were derived from the interval between January 2020 and September 2020. The adjustment of scanning parameters was based on recent simulation studies from data of the herein used scanner regarding feasibility of LD imaging for the purpose of various clinical applications^16,17,21,22^.

Patients were consecutively included if they had multi-detector CT (MDCT) scanning available for a biopsy of the vertebral bone or intervertebral disc according to clinical indications (to diagnose unclear bone lesions/suspected bone tumors or suspected inflammation/spondylodiscitis). Patients were identified in the hospital’s institutional digital picture archiving and communication system (PACS). All eligible patient cases with LD scans were matched according to sex (m/f), age (± 10 years), level of the procedure (cervical, thoracic, or lumbosacral), presence/absence of spinal instrumentation (metallic hardware causing artifacts in imaging data and making the access route to the target structure more demanding), and body diameter (< 20 cm, 20–25 cm, 25–30 cm, and > 30 cm). Patients were excluded if 1) non-diagnostic image quality was present in MDCT data (e.g., due to motion artifacts), or 2) the planned biopsy (including survey, planning, and procedure scans) was not accomplished (e.g., due to incompliance of the patient and preliminary abortion of the exam).

Overall, 96 cases were eligible and considered in this study (48 patients with SD scans and 48 matched patients with LD scans, 34 cases each with intervertebral disc biopsy and 14 cases with vertebral bone biopsy).

### Multi-detector computed tomography

All scans included were performed with the patient in prone position using the same 128-slice MDCT scanner (Ingenuity Core 128; Philips Healthcare, Best, The Netherlands). After performing the scout scan covering the planned site of biopsy according to previous diagnostic imaging, a planning scan of the region to be considered was performed (spot scanning). The acquired scan was used for the purpose of procedure planning, with the interventionalist first selecting the slice allowing for optimal visualization of the access route to the intervertebral disc or vertebral body. During the subsequently performed interventional procedure, sequential scanning was achieved for guidance and surveillance during needle placement using a foot pedal (intermittent scanning, three axial images per shot). By default, images were reconstructed with model-based iterative reconstruction (planning scans; IMR1, Philips Healthcare, Best, The Netherlands) or hybrid iterative reconstruction (periprocedural guidance scans; iDose4, Philips Healthcare, Best, The Netherlands). The parameters for the planning and periprocedural guidance scans are illustrated in Table 1.

**Table 1:** Details of the scanning protocol and image reconstruction for the planning and periprocedural guidance scans using a standard-dose (SD) and low-dose (LD) protocol.

	Periprocedural guidance scan		Planning scan	
	Standard-dose (SD) imaging	Low-dose (LD) imaging	Standard-dose (SD) imaging	Low-dose (LD) imaging
Cycle time (in s)	2.4		6	
No. of cycles	1		1	
Rotation time (in s)	0.75		0.75	
Tube voltage (in kV)	120		120	
Tube current (in mA)	40–67	20–40	40–67	20–40
Estimated exposure (in mAs)	~30–60	~15–20	~30–60	~15–20
Collimation width (in mm)	0.625			
Windowing	L = 750.0 ; W = 2500.0			
Image reconstruction	iDose4	iDose4	IMR1	IMR1

**Figure 1:**
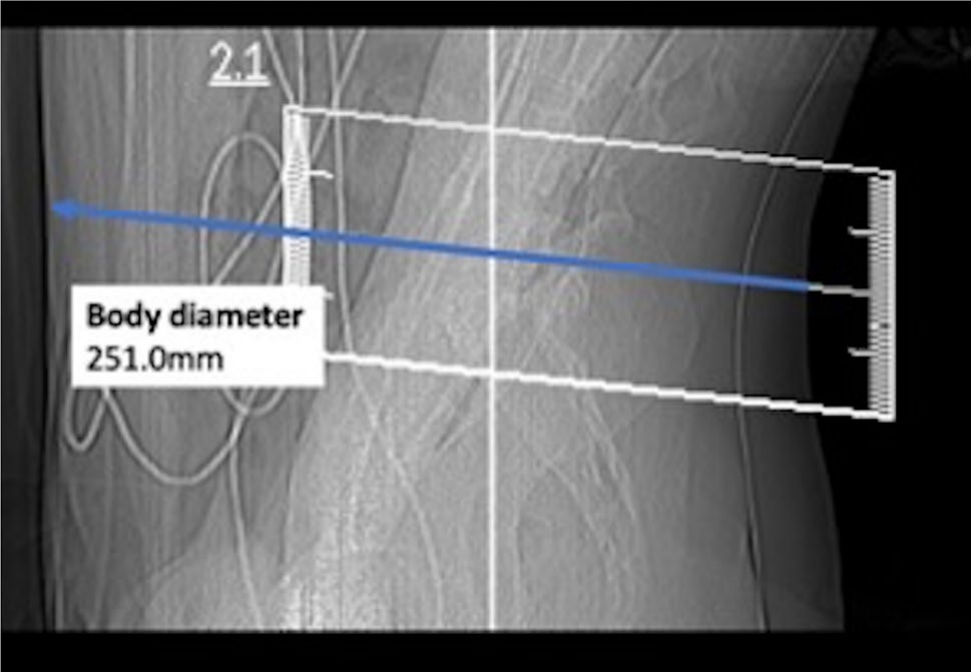
Lateral scout obtained for a planned lumbar intervertebral disc biopsy in a 47-year-old woman with suspected spondylodiscitis at level L4/5. The blue line indicates the antero-posterior body diameter (251.0 mm).

Parameters obtained from SD and LD scanning included the dose length product (DLP), volumetric CT dose index (CTDI_vol_), number of scans required to perform the spine biopsy (periprocedural guidance scans via sequential scanning), and measurements of body diameter. The individual body diameter was measured in the lateral scout scan at the level of the planned intervention and was determined from skin-to-skin surface (Fig. 1)^23^.

### Image evaluation

**Figure 2:**
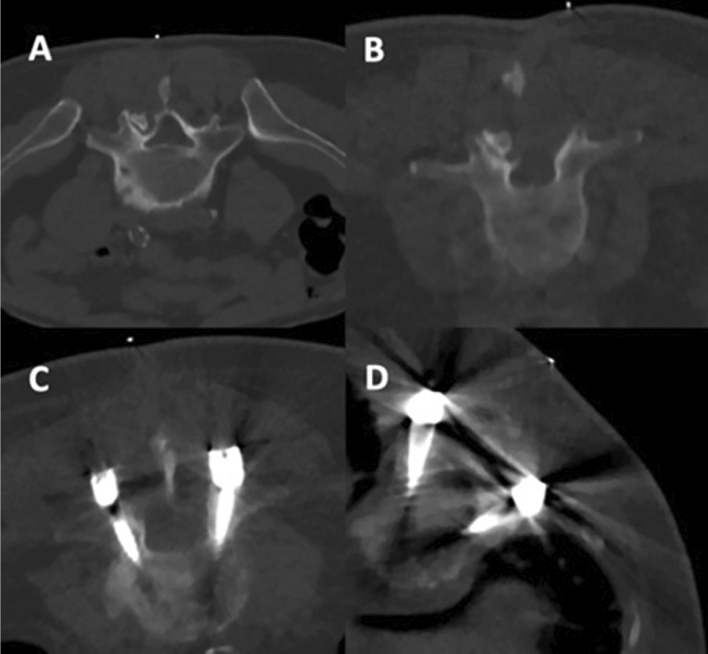
Examples for planning scans for lumbosacral spine biopsies in different patients performed with a low-dose (LD) protocol for scanning, which were rated with perfect (**A**), good (**B**), medium (**C**), and poor (**D**) overall image quality.

After the biopsies, imaging data were transferred to PACS and evaluated with the standard PACS viewer (IDS7; Sectra AB, Linköping, Sweden) by two radiologists (board-certified radiologist with 9 years of experience, reader 1 [R1], and resident in radiology with 2 years of experience, reader 2 [R2]). The readers semi-quantitatively evaluated overall image quality, overall artifacts, image contrast, determination of the target structure (intervertebral disc or vertebral body), and confidence for intervention planning (based on planning scans) and confidence for intervention guidance (based on the sequential scans during performance of the intervention), using 5-point or 3-point Likert scales (Table 2; Fig. 2).

**Table 2:** Semi-quantitative scoring for image evaluation by two readers.

Item	Score				
	1	2	3	4	5
Overall image quality	Very good to perfect	Good to very good	Medium	Poor	No value
Overall artifacts	None	Minimal	Prominent	Major	Severe
Image contrast	Very good to perfect	Good to very good	Medium	Poor	No value
Determination of target structure	Possible	Unclear	Not possible	x	x
Confidence for intervention planning (planning scans before the infiltration)	High	Medium	Low	x	x
Confidence for intervention guidance (sequential scans during the infiltration)	High	Medium	Low	x	x

During evaluations, the readers were strictly blinded to the ratings of each other, and there was an interval of at least 3 weeks between the readings of data of different dose levels. Per image reading round (SD or LD scans), the images of the patients were presented in randomized order. Furthermore, the readers were unaware of the distinct protocol used for scanning per reading round. The readers were presented with images using bone and soft tissue windowing, and they were allowed to manually adjust windowing levels if wanted, starting with a standard output in the PACS viewer (window length = 750.0, width = 2500.0).

**Figure 3:**
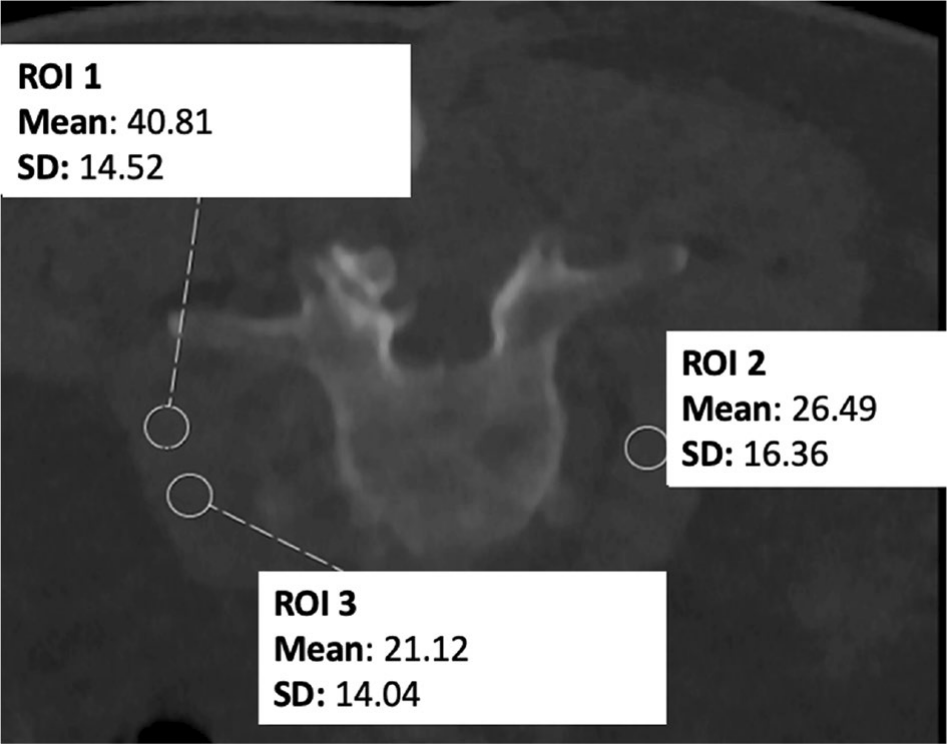
Measurement of image noise for a planned lumbosacral biopsy. Three measurements within the psoas muscle were performed to derive attenuation values from manually placed circular regions of interest (ROIs).

In addition to semi-quantitative rating using Likert scales, quantitative evaluation was performed by measuring image noise at the level of the procedure. This was achieved by manually placing ~10 mm^2^ circular regions of interest (ROIs) and measuring the standard deviation (StDev) of the attenuation in Hounsfield units (HU) in the psoas muscles for lumbosacral and trapezius muscles for cervical interventions^23,24^. In each patient case, three separate measurements were performed (Fig. 3), and the obtained values of the three ROIs were averaged per patient.

### Statistical analysis

GraphPad Prism (version 6.0; GraphPad Software, San Diego, CA, USA) and SPSS (version 28.0; IBM SPSS Statistics for Windows, IBM Corp., Armonk, NY, USA) were used for statistical data analysis. A *p*-value < 0.05 (two-sided) was considered statistically significant.

Descriptive statistics were calculated for the scores assigned by each reader concerning the single items of image evaluation (using Likert scales) and attenuation measurements (in HU) as well as for patient demographics, characteristics of interventions, and dose measurements. Weighted Cohen’s kappa (κ) was calculated to assess inter-reader agreement regarding scorings for overall image quality, overall artifacts, image contrast, determination of the target structure (intervertebral disc or vertebral body), and confidence for intervention planning and intervention guidance during the biopsy. To compare the scores from image evaluation using Likert scales between SD and LD scans, Wilcoxon signed-rank tests were performed per reader. Furthermore, Wilcoxon signed-rank tests were also conducted to compare measures of image noise between SD and LD scans, demographics, and dose characteristics.

### Ethical approval

This retrospective monocentric study involving human participants with a matched pairs design was approved by the ethics committee of the Faculty of Medicine at Technical University of Munich and was in accordance with the ethical standards of the institutional research committee and with the Declaration of Helsinki.

### Informed consent

The requirement for written informed consent was waived by the ethics committee of the Technical University of Munich due to the study’s retrospective design.

## Results

### Patient cohort

Overall, 96 patients were enrolled in this study (48 patients with SD imaging and 48 matched patients with LD imaging). According to matching criteria, in both groups 26 patients were female and 22 patients were male. Indication for intervertebral disc biopsy was made in 34 patients per group, while 14 patients per group underwent biopsy of a vertebral body lesion. Seven patients per group had implants (e.g., after dorsal spinal instrumentation) within the field of view chosen for the intervention. Suspected tumor or metastatic lesions made up the indication for biopsy in 11 patients of the LD group and in 12 patients of the SD group, the remaining patients underwent biopsies due to suspected inflammatory processes (e.g., spondylodiscitis).

**Figure 4:**
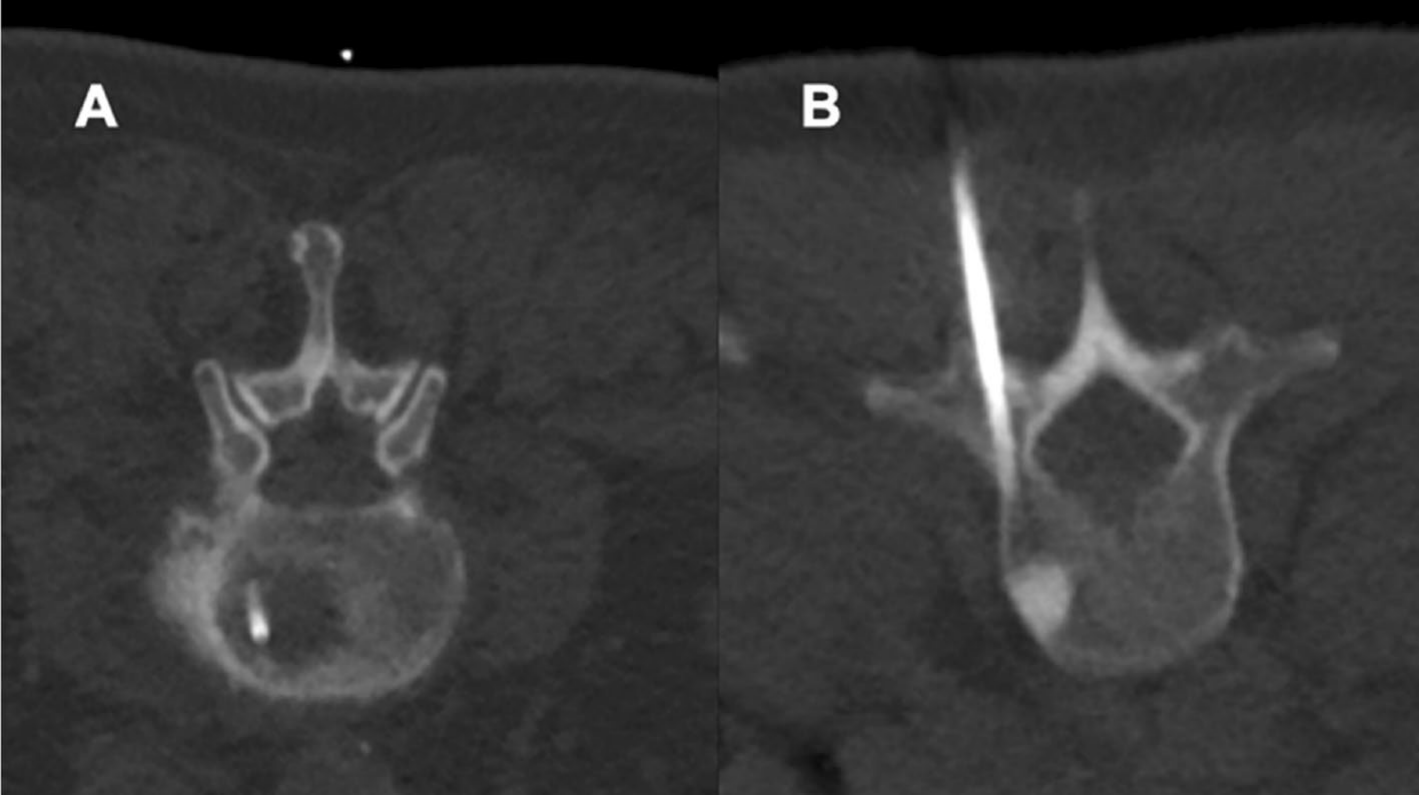
Exemplary patient cases for a L3 bone biopsy for a suspected bone tumor in a 77-year-old male using scanning with standard dose (SD; **A**) and a L1 bone biopsy in a 49-year-old woman with a suspected bone metastasis from known breast cancer using scanning with low dose (LD; **B**). The scans were rated with excellent image quality and high confidence.

**Figure 5:**
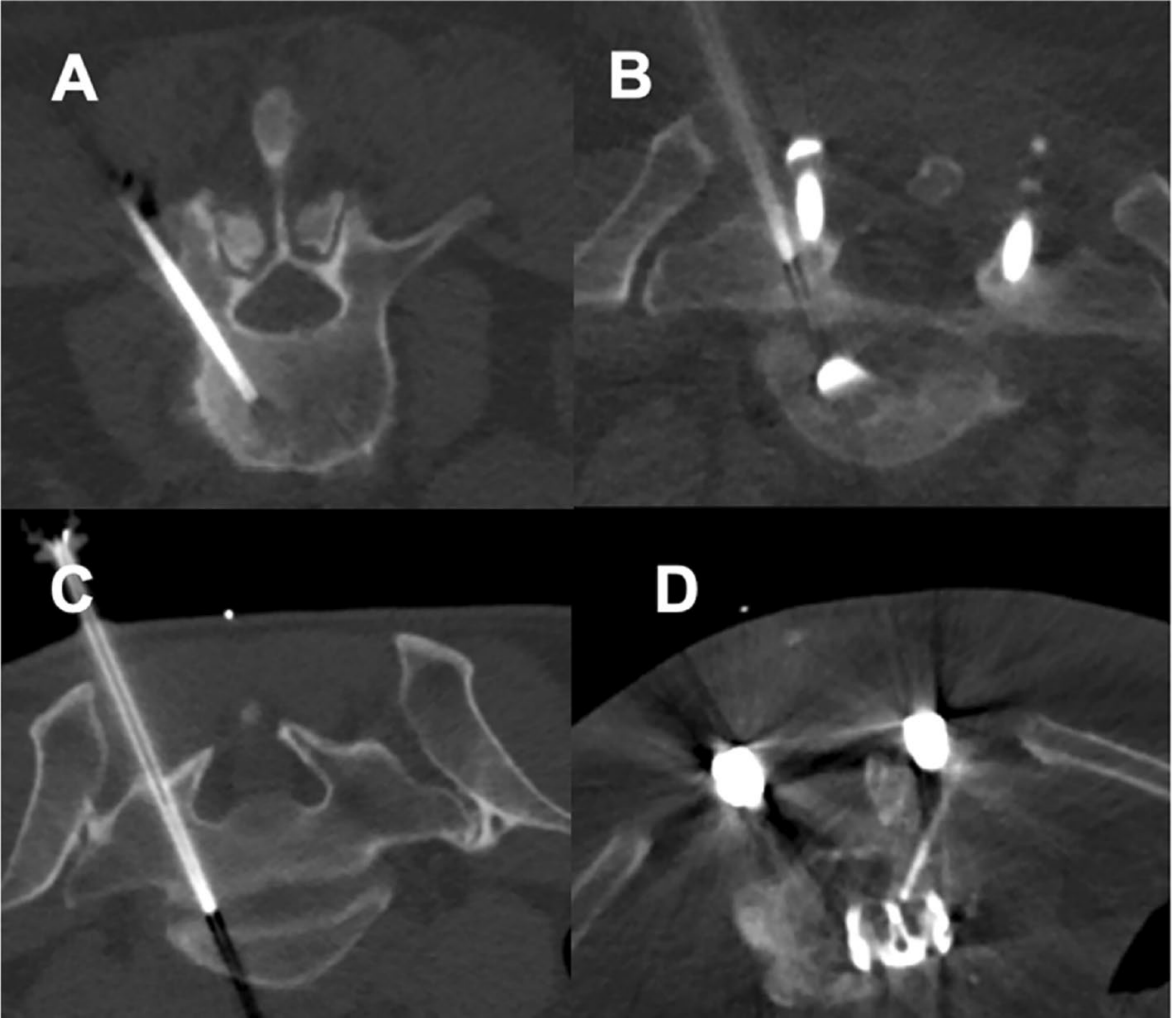
Exemplary patient cases for intervertebral disc biopsies using scanning with standard dose (SD; **A**, **B**) and scanning with low dose (LD; **C**, **D**). The upper row shows a L2/3 intervertebral disc biopsy in a 66-year-old woman (**A**) and a L5/S1 intervertebral disc biopsy in a 58-year-old woman with artifacts due to spinal instrumentation (**B**). The second row shows a L5/S1 intervertebral disc biopsy in a 48-year-old male patient (**C**) and a L4/5 intervertebral disc biopsy in an 80-year-old man with artifacts due to spinal instrumentation (**D**). All patients underwent intervertebral disc biopsy due to suspected spondylodiscitis.

There were no statistically significant differences between patients for SD versus LD scans regarding age, body diameter, or number of sequential scans needed during performance of the intervention (Table 3). Furthermore, no major periprocedural complications (e.g., bleeding) were reported for any of the biopsies performed either with the SD or LD protocol. Figures 4 and 5 depict exemplary patient cases.

**Table 3:** Cohort and procedure characteristics.

	SD	LD	*p* value
Age (in years, mean ± StDev)	67.7 ± 12.9	69.5 ± 12.7	0.65
Body diameter on scout image (in cm, mean ± StDev)	27.7 ± 5.2	25.9 ± 4.2	0.44
Number of scans needed during the intervention (n)	13.1 ± 4.7	13.9 ± 6.4	0.19

### Semi-quantitative evaluation

Image quality, image contrast, the determination of the target structure, and confidence for planning or intervention guidance were rated good to perfect for both SD and LD scans according to evaluations of both readers, without statistically significant differences between SD versus LD scans for these parameters (*p* > 0.05; Tables 4 and 5). Further, inter-reader agreement was at least substantial for the images from intervention planning (range of κ: 0.64–0.90) as well as from intervention guidance (range of κ: 0.72–0.88), except for confidence for intervention guidance with moderate agreement between readers (κ = 0.59; Tables 4 and 5).

**Table 4:** Semi-quantitative scoring for intervention planning scans according to evaluations of two readers (R1 and R2) considering scanning with standard dose (SD) and low dose (LD).

Intervention planning			
	LD	SD	*p* value
Overall image quality			
R1	1 (1–4)	2 (1–4)	0.89
R2	1 (1–4)	2 (1–4)	0.64
Kappa	0.90	0.81	
Overall artifacts			
R1	1 (1–4)	1 (1–4)	0.99
R2	1 (1–4)	1 (1–4)	0.41
Kappa	0.87	0.90	
Image contrast			
R1	1 (1–4)	2 (1–4)	0.17
R2	1 (1–4)	2 (1–3)	0.11
Kappa	0.85	0.72	
Determination of target structure			
R1	1 (1–2)	1 (1–2)	0.99
R2	1 (1–2)	1 (1–2)	0.38
Kappa	0.90	0.77	
Confidence for planning			
R1	1 (1–2)	1 (1–2)	0.25
R2	1 (1–3)	1 (1–3)	0.45
Kappa	0.64	0.74

**Table 5:** Semi-quantitative scoring for periprocedural guidance scans according to evaluations of two readers (R1 and R2) considering scanning with standard dose (SD) and low dose (LD).

Intervention guidance			
	LD	SD	*p* value
Overall image quality			
R1	1 (1–3)	1 (1–3)	0.70
R2	1 (1–4)	1 (1–3)	0.99
Kappa	0.82	0.85	
Overall artifacts			
R1	1 (1–4)	1 (1–3)	0.99
R2	1 (1–4)	1 (1–4)	0.94
Kappa	0.86	0.80	
Image contrast			
R1	1 (1–3)	1 (1–3)	0.82
R2	1 (1–3)	1 (1–3)	0.44
Kappa	0.76	0.72	
Determination of target structure			
R1	1 (1–2)	1 (1–2)	0.63
R2	1 (1–2)	1 (1–2)	0.63
Kappa	0.79	0.88	
Confidence for guidance			
R1	1 (1–2)	1 (1–2)	0.99
R2	1 (1–2)	1 (1–3)	0.25
Kappa	0.85	0.59	

### Radiation exposure

**Figure 6:**
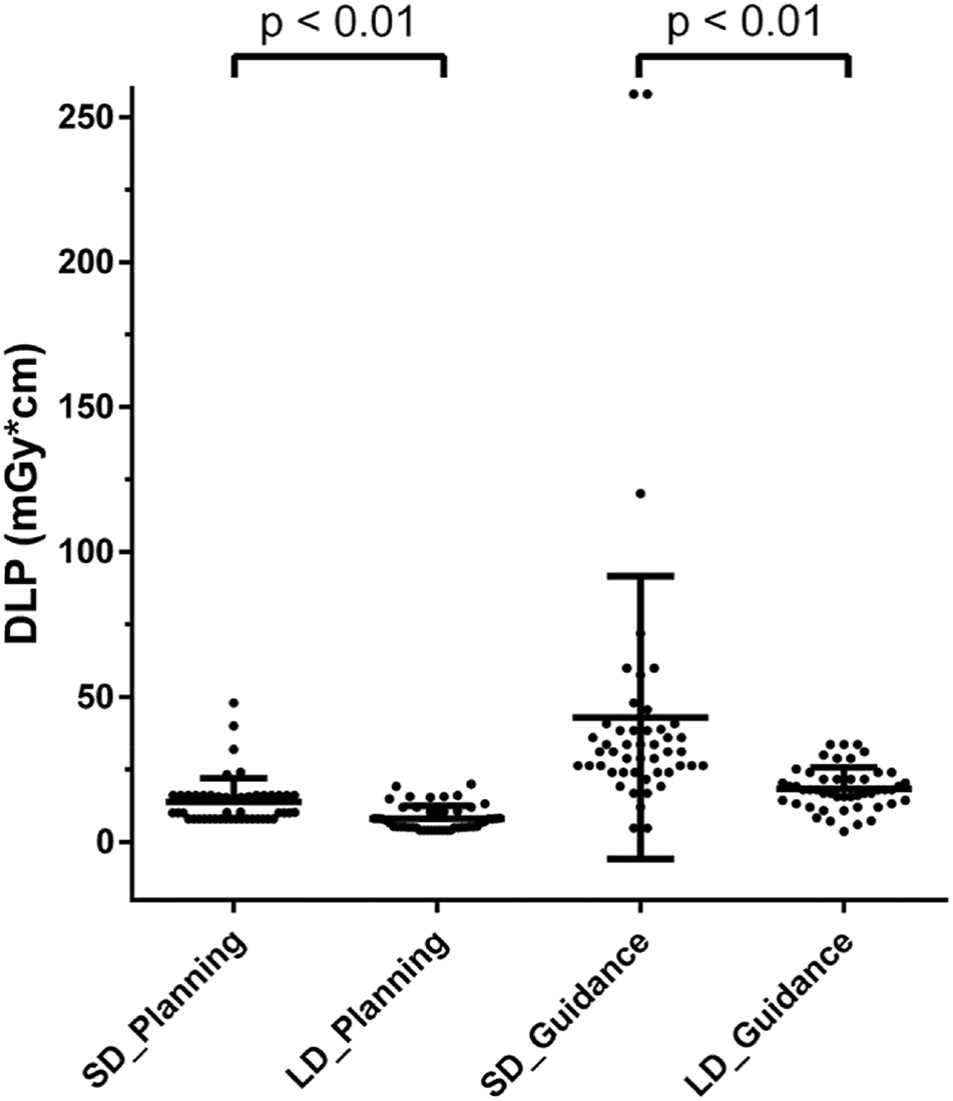
Scatter dot plots with mean ± standard deviation (StDev) for the dose-length product (DLP, in mGy*cm) of planning and periprocedural guidance scans using scanning with standard dose (SD) and low dose (LD).

The DLP was statistically significantly lower for LD scans regarding the planning scans (SD: 13.8 ± 8.2 mGy*cm, LD: 8.1 ± 4.4 mGy*cm, *p* < 0.01) as well as the periprocedural guidance scans (SD: 43.0 ± 48.8 mGy*cm, LD: 18.4 ± 7.3 mGy*cm, *p* < 0.01; Fig. 6). Similarly, the CTDI_vol_ was also statistically significantly lower for LD scans regarding the planning scans (SD: 2.1 ± 0.4 mGy, LD: 1.3 ± 0.8 mGy, *p* < 0.01) as well as the periprocedural guidance scans (SD: 32.4 ± 14.3 mGy, LD: 18.1 ± 7.4 mGy, *p* < 0.01).

### Image noise

Noise according to quantitative evaluation using muscle attenuation values was similar between SD and LD scans performed for planning of the interventional procedure (SD: 14.62 ± 2.83 HU vs. LD: 15.45 ± 3.22 HU, *p* = 0.24).

## Discussion

Lowering the tube current for MDCT can be a simple and effective method for reducing radiation exposure to both the patient and interventionalist regarding CT-guided spine biopsies. In our study, we were able to show that dose reduction for planning and periprocedural guidance scans for intervertebral disc and bone biopsies with MDCT is feasible and can be performed without clinically relevant drawbacks regarding image quality or confidence. The DLP and CTDI_vol_ for LD scans were statistically significantly lower regarding the planning scans as well as for the periprocedural guidance scans. Overall image quality, image contrast, the determination of the target structure for biopsy, and confidence for planning or intervention guidance were rated good to perfect for SD and LD scans, respectively. Overall, noise according to quantitative evaluation using muscle attenuation values was similar between SD and LD scans performed for planning of the interventional procedures.

Imaging guidance for biopsy is a commonly used procedure in patients with findings in need for clarification^8,9^. Yet, concerns with CT guidance relate to potential consequences of ionizing radiation. Hence, there are many efforts to keep radiation exposure as low as reasonably achievable (ALARA)^25–27^. The options available for reaching the goal of low radiation dose in CT are manifold and primarily include adaptions in scanning parameters such as for tube current, tube voltage, slice thickness, patient coverage, number of acquisitions, or length of the procedure^26^. In the course of CT scanning protocol optimizations and introduction of model-based iterative reconstruction, we adjusted the CT protocol based on a former conventional SD protocol to provide LD scanning with reduced radiation exposure. Previously published in-vivo studies demonstrated the utility of LD techniques for a multitude of interventional procedures. Specifically, Meng et al. performed a study with focus on biopsies of lung lesions with a LD protocol (group 1: 120 kV, 200 mA; group 2: 120 kV, 10 mA) and revealed that a reduction of radiation dose and DLP were possible without a relevant loss of diagnostic yield^28^. Despite a considerable reduction of the radiation dose during CT-guided percutaneous lung biopsies by more than 90% (from DLP of 677.5 mGy*cm to 18.3 mGy*cm), Smith et al. found no relevant decrease in technical success or patient safety^25^. Furthermore, especially for pediatric CT scanning, many studies were able to show that CT-guided bone biopsies performed using techniques for lowering radiation exposure can also lead to acceptable image quality and provide similar diagnostic yield compared with SD protocols^29–31^.

A substantial proportion of applied radiation results from performing pre- and post-biopsy scans as they are designed to optimize the visualization of soft tissues for needle guidance and to exclude biopsy-related complications. A review by Sarti et al. showed that up to 90% of the total radiation dose during biopsies was caused by the helical planning scan^32^. In our study, we tried to solve this problem using two different methods. On the one hand, we lowered the applied radiation dose by reductions in tube current based on the former SD protocol. On the other hand, we only scanned the region of interest of the planned CT intervention. From this point of view, pre-biopsy imaging should be carefully reviewed and when possible focused on the definite region of biopsy, together with an optimized radiation dose. This approach is similar to a recent study investigating LD protocols for CT-guided periradicular infiltrations^33^. However, biopsies use hardware for puncture of the target region with comparatively large calibers (e.g., potential risk for more pronounced artifacts especially with LD protocols) that often need to be inserted deeper into tissue (e.g., close to the nerve root in periradicular infiltrations versus biopsies of vertebral bone), making distinct investigations for biopsies necessary in the light of patient safety and sufficient image quality. Further, in a study by Lucey and coworkers, 291 CT-guided interventional procedures without the use of CT fluoroscopy (for percutaneous biopsy, needle aspiration, and percutaneous catheter placement) were performed using a LD protocol with an exposure of 30 mAs and a tube voltage of 120–140 kV for the performance scan^26^. They found that by decreasing the effective tube current and exposure from 180–240 mAs to 30 mAs, radiation to the patient decreased six- to eightfold^26^. At the same time, the technical success rates of biopsies performed at 30 mAs were similar compared to those for biopsies performed with the SD protocol^26^. Yet, in 13 cases where patients underwent biopsies with the new LD protocol, the masses were not clearly identified, thus these procedures were completed at a higher dose^26^. The complication rate of the LD technique was comparable to that of the SD technique in their study^26^. These results are in accordance with our results where a reduction of the radiation dose led to a significant reduction of the DLP regarding the planning scans as well as the interventional guidance scans. Furthermore, the tube current in our study was even lower compared to that previous study.

All intervertebral disc and vertebral body biopsies in our study were performed under CT guidance. Other publications have reported that the use of CT fluoroscopy exposes patients to less radiation than conventional CT-guided procedures^34,35^. Yet, the use of CT fluoroscopy can expose the interventionalist to more radiation than using conventional CT techniques^26^. We believe that the use of LD techniques under CT fluoroscopic guidance may be the optimal way to decrease radiation exposure during procedures. McNamara and coworkers provided a systematic review and meta-analysis about image-guided biopsies conducted in patients with suspected discitis and investigated 14 biopsies performed under CT guidance, as well as 6 studies under fluoroscopic guidance^13^. They concluded that fluoroscopic guidance was associated with a higher yield at 55% compared with CT guidance at 44%, though the difference was not statistically significant^13^.

Recent techniques have set a focus on iterative image reconstruction models such as adaptive statistical iterative reconstruction or model-based iterative reconstruction^16,17,22,36–38^. These algorithms are useful options for further dose reductions, not only in diagnostic CT but also in CT-guided interventions^39–41^. Yet, their availability is currently limited to newer CT scanners, and if used in clinical practice, such approaches are primarily applied for diagnostic CT scanning and less for interventional CT scanning. Prospectively, a wider availability of iterative reconstruction software may translate into an increased comfort for the radiologist while evaluating images with LD regimens during CT-guided interventions and, therefore, potentially result in further reductions of the radiation dose applied to the patients. In our study, all planning scans were reconstructed using model-based iterative reconstruction algorithms, given that model-based iterative approaches may help to increase the visibility of anatomical details whilst facilitating LD protocols^39–41^.

Yet, there are some limitations to this study. First, the study design was retrospective and the study was performed at a single academic institution, implicating that spine and intervertebral disc biopsies were conducted by different interventionalists with different education levels. Therefore, the reproducibility of the results using the new LD protocol cannot be fully assessed in this study. Second, the lack of quantifying artifacts specifically associated with the metallic needle can be considered a limitation, which can lead to restrictions in overall image quality and thus can have impact on periprocedural guidance. Third, despite a different level of experience and education of the two readers (radiologist with 9 years and resident with 2 years of experience), the reading results were very good and similar with only small differences between the readers. This could be a hint for a LD protocol that could have been subject to even further decreases in radiation dose. On the other hand, this could also indicate that dose reduction in CT-guided spine biopsies is an applicable method in clinical practice that does not result in considerably reduced image quality or diagnostic confidence when performed with the herein presented protocol, neither for an experienced radiologist nor a resident with much less experience. Furthermore, it would be difficult to determine whether more experienced radiologists may have contributed to a greater efficiency of the procedure using a LD protocol. As a consequence, there is a potential bias for radiation exposure as well as procedural time and number of scans needed during intervention that is inherent to the study design. Fourth, the reduced dose level was reached by lowering the tube current in combination with model-based iterative reconstruction, but without evaluating other modern approaches to limit radiation exposure, such as sparse sampling that has been successfully applied for spine CT in previous simulation studies^16,17,21,42^. Furthermore, we did not explicitly assess which parameter adjustments contributed the most to radiation dose reductions, and this may need to be solved primarily by phantom or cadaver studies in which more than one scan could be achieved, given that radiation protection is not an issue. However, a recent review concluded that nowadays, considerable dose reductions in spinal CT for various indications can be realized with dose reductions around 50%^43^. Although some novel approaches during image acquisitions have not yet been implemented in commercially available MDCT systems (e.g., sparse sampling), such techniques may become of general interest in the near future^44,45^. Additionally, for improving image quality and reducing radiation dose, deep learning-based reconstruction approaches are promising tools^41,46,47^.

## Conclusion

We demonstrated that a LD imaging protocol combined with advanced image reconstruction for MDCT scanning during planning and performing intervertebral disc or vertebral body biopsies is a viable option, given that radiation exposure to the patient significantly decreased without considerably affecting image quality or the confidence for planning and guidance. Furthermore, despite a significantly lower total cumulative radiation exposure compared with regular-dose CT-guided biopsies, using a LD protocol did not alter the rate of complications. We therefore encourage other centers to consider LD imaging protocols combined with model-based iterative reconstruction as an alternative to conventional protocols for CT-guided spine interventions.

## Data Availability

The datasets used and analysed during the current study are available anonymized from the corresponding author on reasonable request.

## References

[1] Takenaka D (2009). Detection of bone metastases in non-small cell lung cancer patients: Comparison of whole-body diffusion-weighted imaging (DWI), whole-body MR imaging without and with DWI, whole-body FDG-PET/CT, and bone scintigraphy. J. Magn. Reson. Imaging.

[2] Gallucci PM, D'Orazio F (2015). Image guided interventions in spinal infections. Neuroimaging Clin. N. Am..

[3] Rimondi E (2011). Percutaneous CT-guided biopsy of the musculoskeletal system: Results of 2027 cases. Eur. J. Radiol..

[4] Rimondi E (2001). Computerized tomography guided biopsy in the diagnosis of neoplastic and inflammatory lesions of the pelvis. Radiol. Med..

[5] Rimondi E (2008). Percutaneous CT-guided biopsy of the spine: results of 430 biopsies. Eur. Spine J..

[6] Menon VK, Kumar KM, Al Ghafri K (2014). One-stage biopsy, debridement, reconstruction, and stabilization of pyogenic vertebral osteomyelitis. Glob. Spine J.

[7] Sertic M (2019). The efficacy of computed tomography-guided percutaneous spine biopsies in determining a causative organism in cases of suspected infection: A systematic review. Can. Assoc. Radiol. J..

[8] Saifuddin A (2021). Review article: The current status of CT-guided needle biopsy of the spine. Skelet. Radiol..

[9] Yang SY (2018). Percutaneous image-guided spinal lesion biopsies: factors affecting higher diagnostic yield. AJR Am. J. Roentgenol..

[10] Rehm J (2016). CT-guided percutaneous spine biopsy in suspected infection or malignancy: A study of 214 patients. Rofo.

[11] Singh DK (2020). Approach-based techniques of CT-guided percutaneous vertebral biopsy. Diagn. Interv. Radiol..

[12] Peh W (2006). CT-guided percutaneous biopsy of spinal lesions. Biomed. Imaging Interv. J..

[13] McNamara AL (2017). Yield of image-guided needle biopsy for infectious discitis: A systematic review and meta-analysis. AJNR Am. J. Neuroradiol..

[14] Nagayama Y (2021). Deep learning-based reconstruction for lower-dose pediatric CT: Technical principles, image characteristics, and clinical implementations. Radiographics.

[15] Kertesz H (2021). Reducing radiation exposure to paediatric patients undergoing [18F]FDG-PET/CT imaging. Mol. Imaging Biol..

[16] Sollmann N (2021). Low-dose MDCT of patients with spinal instrumentation using sparse sampling: Impact on metal artifacts. AJR Am. J. Roentgenol..

[17] Sollmann N (2021). Low-dose MDCT: Evaluation of the impact of systematic tube current reduction and sparse sampling on the detection of degenerative spine diseases. Eur. Radiol..

[18] Becce F (2013). Computed tomography of the cervical spine: comparison of image quality between a standard-dose and a low-dose protocol using filtered back-projection and iterative reconstruction. Skelet. Radiol..

[19] Lee SH (2017). Diagnostic usefulness of low-dose lumbar multi-detector CT with iterative reconstruction in trauma patients: Acomparison with standard-dose CT. Br. J. Radiol..

[20] Wiesner EL (2018). Percutaneous CT-Guided biopsies of the cervical spine: Technique, histopathologic and microbiologic yield, and safety at a single academic institution. AJNR Am. J. Neuroradiol..

[21] Sollmann N (2019). Multi-detector CT imaging: Impact of virtual tube current reduction and sparse sampling on detection of vertebral fractures. Eur. Radiol..

[22] Sollmann N (2019). Systematic evaluation of low-dose MDCT for planning purposes of lumbosacral periradicular infiltrations. Clin. Neuroradiol..

[23] Amrhein TJ (2016). Reducing patient radiation exposure from CT fluoroscopy-guided lumbar spine pain injections by targeting the planning CT. AJR Am. J. Roentgenol..

[24] Schindera ST (2008). Effect of patient size on radiation dose for abdominal MDCT with automatic tube current modulation: phantom study. AJR Am. J. Roentgenol..

[25] Smith JC (2011). Ultra-low-dose protocol for CT-guided lung biopsies. J. Vasc. Interv. Radiol..

[26] Lucey BC (2007). CT-guided intervention with low radiation dose: feasibility and experience. AJR Am. J. Roentgenol..

[27] Bevelacqua JJ (2010). Practical and effective ALARA. Health Phys..

[28] Meng XX (2013). Comparison of lung lesion biopsies between low-dose CT-guided and conventional CT-guided techniques. Acta Radiol..

[29] Kalra MK (2004). Strategies for CT radiation dose optimization. Radiology.

[30] Natali GL (2016). Paediatric musculoskeletal interventional radiology. Br. J. Radiol..

[31] Patel AS (2013). Radiation dose reduction in pediatric CT-guided musculoskeletal procedures. Pediatr. Radiol..

[32] Sarti M, Brehmer WP, Gay SB (2012). Low-dose techniques in CT-guided interventions. Radiographics.

[33] Paprottka KJ (2022). Low-dose multi-detector computed tomography for periradicular infiltrations at the cervical and lumbar spine. Sci. Rep..

[34] Teeuwisse WM (2001). Patient and staff dose during CT guided biopsy, drainage and coagulation. Br. J. Radiol..

[35] Paulson EK (2001). CT fluoroscopy–guided interventional procedures: Techniques and radiation dose to radiologists. Radiology.

[36] Corcuera-Solano I (2014). Repeated head CT in the neurosurgical intensive care unit: Feasibility of sinogram-affirmed iterative reconstruction-based ultra-low-dose CT for surveillance. AJNR Am. J. Neuroradiol..

[37] Mathieu KB (2014). Radiation dose reduction for CT lung cancer screening using ASIR and MBIR: A phantom study. J. Appl. Clin. Med. Phys..

[38] Flicek KT (2010). Reducing the radiation dose for CT colonography using adaptive statistical iterative reconstruction: A pilot study. AJR Am. J. Roentgenol..

[39] Willemink MJ (2013). Iterative reconstruction techniques for computed tomography Part 1: Technical principles. Eur. Radiol..

[40] Willemink MJ (2013). Iterative reconstruction techniques for computed tomography part 2: Initial results in dose reduction and image quality. Eur. Radiol..

[41] Willemink MJ, Noel PB (2019). The evolution of image reconstruction for CT-from filtered back projection to artificial intelligence. Eur. Radiol..

[42] Mei K (2017). Is multidetector CT-based bone mineral density and quantitative bone microstructure assessment at the spine still feasible using ultra-low tube current and sparse sampling?. Eur. Radiol..

[43] Dieckmeyer, M. *et al*. Computed tomography of the spine: Systematic review on acquisition and reconstruction techniques to reduce radiation dose*.**Clin. Neuroradiol.* (2022). 10.1007/s00062-022-01227-1 PMC10220150 36416936

[44] Chen, B. *et al*. First multislit collimator prototype for SparseCT: Design, manufacturing and initial validation. In *The Fifth International Conference on Image Formation in X-Ray Computed Tomography, Salt Lake City*. (2018).

[45] Koesters, T. *et al*. SparseCT: Interrupted-beam acquisition and sparse reconstruction for radiation dose reduction. In *SPIE Medical Imaging*. SPIE (2017).

[46] Brady SL (2021). Improving image quality and reducing radiation dose for pediatric CT by using deep learning reconstruction. Radiology.

[47] Yeoh H (2021). Deep learning algorithm for simultaneous noise reduction and edge sharpening in low-dose CT images: A pilot study using lumbar spine CT. Korean J. Radiol..

